# Perceived Exercise Barriers and Functional Health Among Older Chinese Women: The Roles of Physical Activity Participation and Community Sports Facility Accessibility

**DOI:** 10.3390/healthcare14172832

**Published:** 2026-09-03

**Authors:** Xinran Cui, Junyi Zheng

**Affiliations:** School of Physical Education, Huazhong University of Science and Technology, 1037 Luoyu Road, Hongshan District, Wuhan 430074, China; m202475617@hust.edu.cn

**Keywords:** older women, perceived exercise barriers, physical activity participation, functional health, community sports facility accessibility, healthy aging

## Abstract

**Highlights:**

**What are the main findings?**

- Perceived Exercise Barriers were associated with lower Basic Activities of Daily Living and reduced Physical Activity Participation among older women in China.
- Physical Activity Participation showed a significant indirect association between Perceived Exercise Barriers and Basic Activities of Daily Living, while Community Sports Facility Accessibility moderated the strength of this indirect association.

**What are the implications of the main findings?**

- Healthy aging strategies for older women should address perceived exercise barriers by supporting sustained Physical Activity Participation rather than relying only on individual health awareness.
- Community sports governance should prioritize accessible, safe, and age-friendly exercise environments, especially for older women facing higher health and socioeconomic vulnerability.

**Abstract:**

**Background:** Physical Activity Participation is closely associated with functional health among older women, but this participation is often constrained by multiple perceived barriers. The aim of this study was to examine the association between Perceived Exercise Barriers (PEB) and Basic Activities of Daily Living (BADL) among older women in China, investigate the indirect association of Physical Activity Participation (PAP) in this relationship, and examine the moderating role of Community Sports Facility Accessibility (CSFA) in the association between PEB and PAP. **Methods:** Using data from the 2023 China Longitudinal Aging Social Survey (CLASS), this cross-sectional study included 5027 women aged 60 years and above. Perceived Exercise Barriers were operationalized as the sum of 18 dichotomous barrier indicators. Basic Activities of Daily Living were measured by summing 11 items assessing daily living functions. Physical Activity Participation was calculated by summing scores across three dimensions: exercise frequency, duration per session, and exercise intensity. Community Sports Facility Accessibility was measured according to the number of available types of community sports resources. A conditional indirect association model was estimated using the SPSS PROCESS macro, and confidence intervals were obtained through the bootstrap method (5000 resamples). **Results:** Perceived Exercise Barriers were significantly and negatively associated with Basic Activities of Daily Living (*B* = −0.103, *p* < 0.001) and Physical Activity Participation (*B* = −0.471, *p* < 0.001) among older women. Physical Activity Participation showed a significant but relatively small indirect association between Perceived Exercise Barriers and Basic Activities of Daily Living (indirect effect = −0.034, 95% CI −0.044 to −0.024). Furthermore, Community Sports Facility Accessibility significantly moderated the association between Perceived Exercise Barriers and Physical Activity Participation (interaction term *B* = 0.094, *p* < 0.001). Higher levels of facility accessibility were associated with a weaker negative association between Perceived Exercise Barriers and Physical Activity Participation, accompanied by a reduced absolute value of the conditional indirect association. **Conclusions:** This study identified a potential behavioral pathway linking Perceived Exercise Barriers, Physical Activity Participation, and Basic Activities of Daily Living among older women, and demonstrated the conditional role of Community Sports Facility Accessibility in this association. The findings suggest that improving functional health among older women may require attention to individual perceived barriers, behavioral participation, and supportive community environments.

## 1. Introduction

Population aging has become a major challenge for global public health and social governance. By the end of 2025, the population aged 60 years and above in China reached 323 million, accounting for 23.0% of the total population, while the population aged 65 and above reached 224 million, making up 15.9% [1]. As population aging continues to deepen, maintaining the functional health of older adults through lifestyle intervention has become a core topic in healthy aging practice [2]. The World Health Organization defines “healthy aging” as the process of developing and maintaining the functional ability that enables well-being in older age [3]. Within this framework, functional health is related not only to older adults’ independence in daily life but also to family caregiving demands and the allocation of community public service resources. Compared with the general older population, older women are more susceptible to multiple factors, such as chronic disease burden, functional decline risks, family caregiving responsibilities, and socio-economic resource constraints. Therefore, the maintenance of their functional health carries even more prominent significance in public health and public management.

Against the background of healthy aging shifting from “lifespan extension” toward “functional maintenance,” the preservation of Basic Activities of Daily Living among older women depends not only on individual physical conditions but is also closely associated with health-related behavioral participation and environmental conditions [4,5]. Physical activity, as an important lifestyle behavior for maintaining physical function and promoting healthy aging, provides an important perspective for understanding differences in functional health among older women [6]. Meanwhile, Physical Activity Participation among older women is not determined solely by individual willingness but emerges from the combined influence of individual perceptions, behavioral choices, and community environmental conditions [7]. Therefore, it is necessary to incorporate perceived barriers, Physical Activity Participation, and community resource conditions into an integrated public health and community governance framework to examine the associated factors and association patterns underlying functional health among older women in China.

However, existing studies have rarely comprehensively examined the associations among individual Perceived Exercise Barriers, Physical Activity Participation, and community sports resources within an integrated analytical framework. Particularly among older women, empirical evidence remains limited regarding the associations between Perceived Exercise Barriers, Physical Activity Participation, and Basic Activities of Daily Living, as well as the potential conditional role of community sports resources. Therefore, this study used data from the 2023 China Longitudinal Aging Social Survey (CLASS) and focused on women aged 60 years and above in China to examine the associations among Perceived Exercise Barriers (PEB), Physical Activity Participation (PAP), and Basic Activities of Daily Living (BADL), and to test the moderating role of Community Sports Facility Accessibility (CSFA) in the association between PEB and PAP. By integrating individual perceived barriers, behavioral participation, and community environmental factors, this study provides a framework for analyzing functional health among older women and offers empirical evidence for community sports resource allocation and public health governance in the context of healthy aging.

## 2. Literature Review and Hypotheses Development

### 2.1. Perceived Exercise Barriers and Basic Activities of Daily Living

Perceived barriers are a core concept in health behaviour research, referring to individuals’ subjective cognitive appraisal of the costs, potential risks, and resource constraints involved in adopting a health behaviour [8]. Existing studies have demonstrated that insufficient physical exercise among older adults is not simply a matter of willingness, but is closely related to their comprehensive assessment of activity risks, time costs, and resource availability [9,10]. Recent systematic reviews on physical activity among community-dwelling older adults suggest that perceived barriers mainly stem from three domains: individual, social, and environmental. At the individual level, pain, fatigue, chronic diseases, declining physical capacity, and concerns about injury and falls may reduce older adults’ evaluations of the safety and feasibility of exercise [11,12]. At the social level, a lack of family or peer support, caregiving responsibilities, and insufficient discretionary time may weaken the practical conditions for sustained participation in physical activity [13]. At the environmental level, factors such as inadequate venues and facilities, inconvenient transportation, high participation costs, and low perceived community safety may increase both the actual cost and psychological burden of participating in physical activities [14,15]. Therefore, Perceived Exercise Barriers are not a single psychological factor but a composite constraint cognition formed by older women while assessing their own physical conditions, social support, and external environmental resources. Although existing studies have demonstrated the association between these barriers and lower levels of Physical Activity Participation, direct evidence regarding their association with Basic Activities of Daily Living among older women remains limited. When older women perceive physical exercise and daily activities as high-risk, burdensome, or unfeasible behaviours, their participation in functional activities such as walking, stair climbing, outdoor activities, and group exercise may decrease. Lower levels of activity participation are generally accompanied by poorer physical function and a higher risk of functional limitations [16,17]. Accordingly, this study proposes the following hypothesis:

**H1.**  *Perceived Exercise Barriers are significantly and negatively associated with Basic Activities of Daily Living among older women.*

### 2.2. The Indirect Association of Physical Activity Participation

Physical Activity Participation may serve as a behavioral pathway underlying the association linking perceived barriers to Basic Activities of Daily Living. The Capability–Opportunity–Motivation–Behaviour (COM-B) model suggests that the occurrence of health behaviour depends on whether an individual possesses the corresponding capability, opportunity, and motivation [18]. Therefore, older adults’ Physical Activity Participation is not determined solely by their willingness to exercise, but is constrained by multiple factors simultaneously. For older women, when physical discomfort, heavy family caregiving burdens, insufficient social support, or unfavourable environmental conditions are perceived as exercise barriers, both their willingness to participate in physical activity and their actual participation behaviour may be inhibited [9,19]. Thus, higher levels of Perceived Exercise Barriers are expected to be associated with a lower likelihood of Physical Activity Participation among older women.

Second, Physical Activity Participation is an important health-related behavior that is closely associated with Basic Activities of Daily Living among older adults. Regular physical exercise helps delay functional decline in Activities of Daily Living (ADL) and maintain independence in daily life [20,21]. Numerous longitudinal evidence shows that higher physical activity levels significantly reduce the risk of ADL decline, whereas insufficient exercise increases the probability of functional impairment [22]. Similar findings have been reached by existing research within the Chinese older population [23]. Moreover, studies based on CLASS data have also shown that appropriate or sufficient physical activity exerts positive impacts on improving both Basic Activities of Daily Living (BADL) and Instrumental Activities of Daily Living (IADL) among older adults [24].

In summary, Perceived Exercise Barriers may not only affect older women’s Physical Activity Participation but may also be associated with functional health through health behaviour. As an important health promotion approach for older adults, Physical Activity Participation reflects the association between perceived barriers and behavioral choices, and is further associated with Basic Activities of Daily Living. Accordingly, this study proposes the following hypothesis:

**H2.**  *Physical Activity Participation has an indirect association between Perceived Exercise Barriers and Basic Activities of Daily Living among older women.*

### 2.3. The Moderating Role of Community Sports Facility Accessibility

Perceived Exercise Barriers reflect older women’s subjective cognitive appraisal of exercise costs, physical risks, and participation feasibility, representing an individual-level factor [8]. However, according to the Social Ecological Model (SEM), health behaviour is shaped not only by individual cognition but also by external factors such as family, community, physical environment, and public services [7,25].

Community Sports Facility Accessibility is considered an important environmental factor that is closely associated with Physical Activity Participation among older women [26]. For older women whose activity radius is relatively limited and who place greater emphasis on safety and convenience, community fitness facilities and public activity venues may reduce travel costs and participation thresholds, thereby increasing physical activity opportunities [27]. Existing literature confirms that environmental factors such as exercise venues, open spaces in parks, walkability, traffic safety, and community sports facilities are all significantly correlated with the physical activity levels of older adults [28,29]. This suggests that Community Sports Facility Accessibility is not an individual motivation but an important environmental resource that facilitates or constrains Physical Activity Participation. Consequently, the influence of individual cognition on behaviour is often conditioned by external environmental circumstances [30]. Under similar levels of perceived barriers, convenient and safe community sports facilities may weaken the negative association of perceived barriers on Physical Activity Participation among older women. Conversely, when community sports resources are insufficient or inconvenient to use, this negative effect may be further strengthened. Accordingly, this study proposes the following hypothesis:

**H3.**  *Community Sports Facility Accessibility moderates the association between Perceived Exercise Barriers and Physical Activity Participation among older women. Specifically, higher Community Sports Facility Accessibility is associated with a weaker negative association between Perceived Exercise Barriers and Physical Activity Participation, whereas lower accessibility is associated with a stronger negative association.*

**Figure 1:**
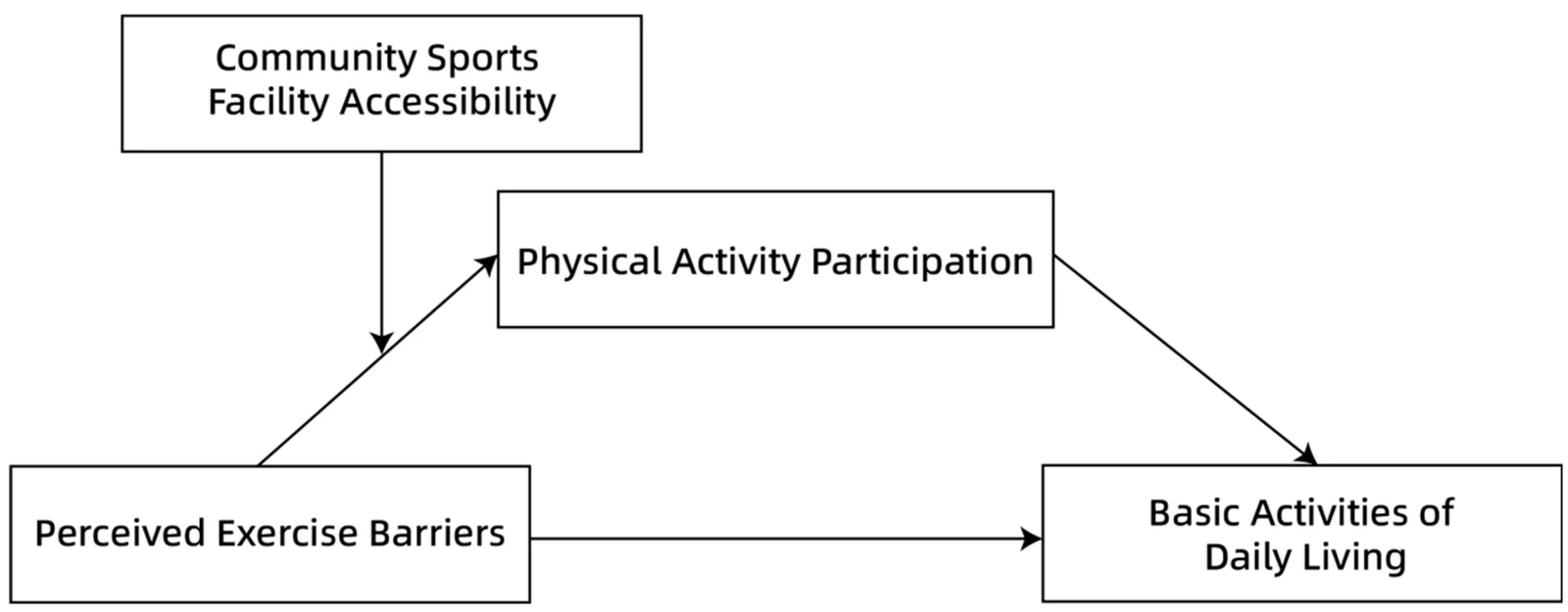
Conceptual framework.

The conceptual framework and hypothesized relationships are illustrated in Figure 1.

## 3. Materials and Methods

### 3.1. Data Source and Study Design

This cross-sectional study used data from the 2023 China Longitudinal Aging Social Survey (CLASS). CLASS is a nationwide social survey of the older population in China that adopts a stratified multistage probability sampling design and covers older residents living in urban and rural communities across 28 provinces (autonomous regions and municipalities directly under the central government). The CLASS 2023 dataset initially included 11,670 respondents. The present study focused on female respondents aged 60 years and above at the time of the survey. According to the inclusion criteria, respondents whose birth-year information did not meet the age eligibility criteria were first excluded (n = 22), followed by the exclusion of male respondents (n = 6042), resulting in 5606 eligible older women. Subsequently, 579 respondents with missing values in key study variables or covariates were excluded using complete-case analysis, resulting in a final analytical sample of 5027 older women. Because CLASS 2023 does not provide information regarding the specific reasons for item non-response or the missing-data process, the missing-data mechanism could not be directly determined based on the available data. Therefore, it was not possible to ascertain whether the missing data followed a Missing Completely At Random (MCAR), Missing At Random (MAR), or Missing Not At Random (MNAR) mechanism.

### 3.2. Measures

#### 3.2.1. Dependent Variable: Basic Activities of Daily Living (BADL)

The dependent variable was Basic Activities of Daily Living (BADL). This variable was constructed from 11 items in the CLASS 2023 questionnaire, including making phone calls, grooming, dressing, bathing, eating, taking medication, bladder and bowel control, toileting, bed-to-chair transfer, and indoor walking [31]. Each item was scored on a three-point Likert scale in the original questionnaire: 1 = no help needed, 2 = some help needed, and 3 = completely unable to do. To maintain directional consistency, reverse scoring was applied to each item so that a higher score represents better Basic Activities of Daily Living. The scores of the 11 items were summed, yielding a total score ranging from 11 to 33.

#### 3.2.2. Core Independent Variable: Perceived Exercise Barriers (PEB)

The core independent variable was Perceived Exercise Barriers (PEB). This variable was derived from the multiple-choice question in the CLASS 2023 questionnaire: “What are the main factors that affect your participation in physical exercise?” The items covered personal, family, and social dimensions. To avoid measurement overlap with the moderator, Community Sports Facility Accessibility, the option “lack of venues and facilities” in the social dimension was excluded. In addition, to ensure comparability across barrier items, the “other” option in each dimension was also excluded.

It should be noted that PEB in this study was not intended to measure a single latent psychological trait. Instead, it was constructed as a cumulative count index based on the barriers actually reported by respondents. Similar barrier-count approaches have been applied in physical activity research, in which the number of reported barriers is used to characterize overall participation constraints rather than assuming that different barrier items reflect a common underlying construct [32,33]. Accordingly, this study adopted an equal-weighted counting approach, assigning one point for each reported barrier and summing the scores across all items. The final PEB index consisted of 18 barrier items, including eight personal barriers, five family-related barriers, and five social barriers. Each item was coded dichotomously (0 = not selected, 1 = selected). The summed score was used to generate the PEB count index, ranging from 0 to 18, with higher scores indicating that respondents reported a greater number of barriers to Physical Activity Participation.

#### 3.2.3. Key Behavioral Variable: Physical Activity Participation (PAP)

The key behavioral variable was Physical Activity Participation (PAP). It was constructed as a relative Physical Activity Participation index based on three items from CLASS 2023: exercise frequency, duration per exercise session, and exercise intensity. Exercise frequency was scored from 1 to 9, duration per session from 1 to 4, and intensity from 1 to 3. The three scores were summed to obtain the PAP, ranging from 3 to 16. 

This scoring approach retained the original ordinal coding of CLASS items without assigning additional theoretical weights. Because the response ranges of the three items differed, exercise frequency contributed relatively more to the numerical value of the total score. Therefore, this index was used only to distinguish relative differences in PAP within the sample; higher scores indicate higher relative PAP levels rather than estimated weekly physical activity volume or achievement of the WHO 2020 physical activity recommendations. Existing physical activity assessment instruments also differ in their indicator composition and scoring approaches. For example, the Physical Activity Scale for the Elderly (PASE) [34], the EPIC Physical Activity Questionnaire (EPIC-PAQ) [35], and the Godin-Shephard Leisure-Time Physical Activity Questionnaire [36] employ different forms of composite scoring approaches. Accordingly, this study retained the original response gradients of CLASS items for composite scoring but did not interpret the dimensions as having theoretically validated fixed weights.

To eliminate differences in measurement scales and improve the comparability of regression coefficients, the core continuous variables, including Basic Activities of Daily Living, Perceived Exercise Barriers, and Physical Activity Participation, were standardized using Z-scores [37]. Descriptive statistics report the original scores of each variable to reflect their actual distribution, whereas standardized variables were utilized in the regression, indirect association analysis, and conditional indirect association analysis [38,39].

#### 3.2.4. Moderating Variable: Community Sports Facility Accessibility (CSFA)

The moderating variable was Community Sports Facility Accessibility (CSFA). This variable was derived from the CLASS 2023 question “Does your community have the following activity venues or facilities?” The two selected options were “fitness venues/facilities” and “outdoor activity spaces”, each coded as 0 = no and 1 = yes. These two items were summed to generate the CSFA score, ranging from 0 to 2. Higher scores indicate that respondents reported a greater number of community sports facility resource types. In this study, CSFA was incorporated into the model as a resource-type count indicator. The original 0–2 scoring scheme was retained, and no Z-score standardization was applied. Mean centering was performed only in the conditional indirect association analysis to facilitate the estimation and interpretation of the interaction term. Treating CSFA as a count-based measure in the moderation model aimed to examine whether the association between Perceived Exercise Barriers and Physical Activity Participation differed across levels of community sports resource availability.

#### 3.2.5. Covariates

With reference to existing literature, this study included age, number of chronic diseases, educational attainment, marital status, household registration type, and individual annual income as covariates [23,40,41]. Age was measured by respondents’ actual age at the time of the survey. The number of chronic diseases was the count of chronic disease types reported by the respondent. Educational attainment was ordinally coded from “illiterate” to “bachelor’s degree and above”. Marital status was coded as 0 = no spouse and 1 = with spouse. Household registration type was coded as 0 = agricultural household registration and 1 = non-agricultural household registration. Individual annual income was ordinally assigned into categories: below 2000 RMB, 2000–6000 RMB, 6000–10,000 RMB, and above 10,000 RMB.

### 3.3. Statistical Analysis

Data processing and statistical analysis were conducted using IBM SPSS Statistics 27.0. Missing data were handled using a complete-case analysis approach, in which respondents with missing values in the main study variables or covariates were excluded from subsequent statistical analyses. Among the 5606 female respondents who met the age and sex eligibility criteria, 579 (10.3%) were excluded due to missing information on relevant variables, resulting in a final analytical sample of 5027 participants. To assess potential selection bias arising from complete-case exclusion, this study further compared differences in age, number of chronic diseases, educational attainment, marital status, and household registration type between the complete-case group and the excluded group. Independent-samples *t* tests were used for continuous variables, while Pearson’s *χ*^2^ test was applied for categorical variables, with corresponding effect sizes reported. Detailed comparisons between the complete-case group and the excluded group are provided in Supplementary Material S2.

Categorical variables were presented as frequencies and percentages, while continuous variables were described using means, standard deviations, minimum values, and maximum values. Considering that the main variables in this study included count variables and ordinal categorical variables, Spearman rank correlation analysis was conducted to examine associations among variables. Based on the median value of the original PAP score (4 points), the sample was divided into a lower PAP group (≤4 points) and a higher PAP group (>4 points). Differences in the distribution of categorical variables between groups were examined using Pearson’s *χ*^2^ tests. Differences in BADL scores between groups were assessed using the Mann–Whitney *U* test, with the effect size *r* reported. Furthermore, BADL scores of 33 points were defined as “complete independence”, while scores below 33 points were defined as “functional limitations”. Multivariable logistic regression was then performed to estimate adjusted odds ratios (aORs) and 95% confidence intervals (CIs) for group comparisons.

Ordinary least squares (OLS) regression was used to examine the association between PEB and BADL. Before conducting OLS regression analyses, model diagnostics were performed to assess multicollinearity, residual independence, and residual distribution characteristics, including the variance inflation factor (VIF), residual distribution plots, normal probability plots, and residual scatter plots. Detailed diagnostic results and plots are provided in Supplementary Material S3. Model 1 included only PEB, while Model 2 further adjusted for covariates including age, number of chronic diseases, educational attainment, marital status, household registration type, and personal annual income. Given the apparent ceiling effect in BADL scores, an upper-censored Tobit model was additionally applied to re-estimate the fully adjusted model. Furthermore, supplementary analyses were conducted using self-rated health as an alternative health outcome. Subgroup analyses were performed according to age, chronic disease burden, income, education, household registration type, and marital status. Differences across subgroups were further examined by including interaction terms between PEB and age groups or income groups.

Indirect association and conditional indirect association analyses were conducted using the Hayes PROCESS Macro v4.2. Model 4 was used to examine the indirect association of PAP in the association between PEB and BADL, while Model 7 was used to examine the moderating role of CSFA in the association between PEB and PAP. All related models were adjusted for the aforementioned covariates. The indirect effect and the index of moderated indirect effect were estimated using 5000 bootstrap resamples, and statistical significance was determined based on whether the 95% confidence interval (CI) included zero. Sensitivity analyses were further conducted by re-estimating PROCESS Models 4 and 7 using dichotomized BADL with a logistic Y-model. In addition, CSFA was treated as a categorical variable and dummy-coded, and PROCESS Model 1 was used to re-examine the interaction between PEB and CSFA to assess the robustness of the linear specification of CSFA. All statistical tests were two-tailed, with the significance level set at *p* < 0.05. This study was reported in accordance with the STROBE guidelines for cross-sectional studies, and the complete checklist is provided in the Supplementary Material S1.

## 4. Results

### 4.1. Descriptive Statistics and Correlations

A total of 5027 women aged 60 years and above were included in the final analysis. Among the 5606 respondents who met the inclusion criteria, 576 had missing information on personal annual income, 6 had missing self-rated health information, and 3 had missing values for both variables. After applying complete-case analysis and excluding 579 respondents with the aforementioned missing information, the final analytical sample consisted of 5027 participants. No missing values were observed for the other major study variables. Compared with the excluded group, participants in the complete-case group were older on average and had a greater number of chronic diseases. Significant differences were also observed between the two groups in the distributions of educational attainment, marital status, and household registration type (all *p* < 0.001; see Supplementary Material S2). The differences between groups were relatively more pronounced for the number of chronic diseases and educational attainment, whereas differences in age, marital status, and household registration type were relatively smaller. These findings indicate that the complete-case group and excluded group differed in some observable characteristics.

Table 1 and Table 2 report the sample characteristics and descriptive statistics of the main variables. The mean age of participants was 71.48 years. Regarding the main variables, the mean BADL was 32.61, the mean PEB was 3.18, and the mean PAP was 6.14. Regarding CSFA, 40.16% of respondents lived in communities without relevant sports resources, 52.28% lived in communities with one type of sports resource, and only 7.56% lived in communities with two types of sports resources.

Table 3 presents the correlation analysis of the core variables. PEB was significantly and negatively correlated with BADL (*ρ* = −0.105, *p* < 0.001) and PAP (*ρ* = −0.386, *p* < 0.001). PAP was significantly and positively correlated with BADL (*ρ* = 0.091, *p* < 0.001), providing preliminary evidence for subsequent regression and conditional association analyses. In addition, CSFA showed a very weak negative association with BADL (*ρ* = −0.029, *p* < 0.05). Although this correlation reached statistical significance in the large sample, its practical significance was limited.

### 4.2. Comparison by Physical Activity Participation Level

To further examine the group characteristics and BADL differences among older women with different levels of PAP, participants were divided into a lower PAP group (≤4 points, n = 2609) and a higher PAP group (>4 points, n = 2418) based on the median value of the original PAP score (4 points). As shown in Table 4, significant differences were observed between the two groups in educational attainment, marital status, household registration type, and personal annual income (all *p* < 0.001). The Mann–Whitney *U* test showed that the higher PAP group had a higher overall rank of BADL scores than the lower PAP group (*U* = 2,995,748, *p* < 0.001), although the effect size was small (*r* = 0.078). Moreover, the median BADL score was 33 in both groups, with an interquartile range of 0, indicating a substantial ceiling effect in BADL scores. After BADL was further dichotomized and all covariates were adjusted, multivariable logistic regression showed that the odds of being completely independent were 1.734 times higher in the higher PAP group than in the lower PAP group (aOR = 1.734, 95% CI 1.437–2.093, *p* < 0.001).

### 4.3. Hierarchical OLS Regression Analysis

Table 5 reports the results of the hierarchical OLS regression analyses examining the association between PEB and BADL. In Model 1, which included only PEB, PEB was significantly and negatively associated with BADL (*B* = −0.104, *p* < 0.001). After further adjusting for age, number of chronic diseases, educational attainment, marital status, household registration type, and personal annual income in Model 2, the negative association remained significant (*B* = −0.103, *p* < 0.001). The *R*^2^ of Model 2 was 0.031, indicating that the model explained a limited proportion of the variance in BADL. OLS regression diagnostics showed that the VIF values for all predictors ranged from 1.041 to 1.403, suggesting no substantial multicollinearity. However, the standardized residual normal probability (P-P) plot indicated deviations from normality, suggesting that the normality assumption of OLS residuals was not fully satisfied. Further examination of the BADL score distribution showed that 4437 of the 5027 participants (88.3%) achieved the maximum score of 33 points, indicating a pronounced ceiling effect in BADL scores and limiting the applicability of conventional OLS regression.

Accordingly, the OLS results were treated as baseline estimates, and a Tobit model with a right-censoring limit of 33 points was applied as a sensitivity analysis. After adjusting for the same covariates, the Tobit model showed that PEB remained significantly and negatively associated with BADL (*B* = −2.561, 95% CI −3.075 to −2.047, *p* < 0.001). Because the Tobit model used the original BADL scores, whereas the hierarchical OLS models used standardized BADL scores, the regression coefficients from the two models should not be directly compared. However, the direction and statistical significance of the associations were consistent across models. Overall, both the hierarchical OLS regression and the right-censored Tobit sensitivity analysis indicated a negative association between PEB and BADL, providing support for H1.

### 4.4. Additional Analysis: Self-Rated Health as an Alternative Health Outcome

To further examine whether the association between PEB and health status extended to other health dimensions, an additional analysis was conducted using self-rated health as an alternative health outcome. Self-rated health was derived from respondents’ subjective evaluation of their own health status in CLASS 2023 and was reverse-coded so that higher scores indicated better health status. As shown in Table 6, PEB remained significantly and negatively associated with self-rated health (Model 1: *B* = −0.219, *p* < 0.001; Model 2: *B* = −0.205, *p* < 0.001), with the direction of association consistent with that observed in the primary model. These findings suggest that the negative association between PEB and health status among older women was not limited to functional health indicators but showed a similar direction across different health dimensions.

### 4.5. Subgroup Analysis

Table 7 presents the results of subgroup analyses across different population groups. The results showed that PEB was significantly and negatively associated with BADL across subgroups stratified by age, chronic disease burden, income, education, household registration type, and marital status, with consistent directions of association. Further comparisons indicated that the coefficients across age groups showed an increasing trend with age. Specifically, the negative coefficient was relatively larger among older women aged 80 years and above. However, the difference between age groups was not statistically significant (*F* (2, 5016) = 0.75, *p* = 0.471). Therefore, this finding only reflects descriptive differences and does not provide sufficient evidence for significant heterogeneity across age groups. For income groups, the negative coefficient was numerically largest among participants with an annual income below 2000 yuan. However, the overall interaction effect across income groups did not reach the predefined statistical significance level (*F* (3, 5014) = 2.52, *p* = 0.056). Therefore, overall income-related heterogeneity was not inferred based on this result. In addition, the negative associations appeared relatively stronger among participants with lower educational attainment, agricultural household registration, and no spouse, whereas the associations were relatively similar across groups with different chronic disease burdens. Because these subgroup differences were not further examined using formal interaction tests, they are presented only descriptively and should not be interpreted as statistically significant differences in subgroup effects.

### 4.6. Indirect Association Analysis

The results of PROCESS Model 4 are presented in Table 8. PEB was significantly and negatively associated with PAP (*B* = −0.471, *p* < 0.001), while PAP was significantly and positively associated with BADL (*B* = 0.072, *p* < 0.001). Bootstrap analysis further showed that PEB had a significant indirect association with BADL through PAP (indirect effect = −0.034, 95% CI −0.044 to −0.024). It should be noted that the Y-model in the primary analysis was estimated using OLS, whereas BADL showed a pronounced ceiling effect. Therefore, the b path, c′ path, and the resulting indirect effect inherit the limitations associated with this model specification. When Model 4 was re-estimated using dichotomized BADL with a logistic Y-model, the indirect effect remained significant (effect = −0.098, 95% bootstrap CI −0.147 to −0.051). Thus, H2 was supported.

### 4.7. Conditional Indirect Association Analysis

PROCESS Model 7 was used to examine whether CSFA moderated the association between PEB and PAP. As shown in Table 9, the interaction term between PEB and CSFA was significantly and positively associated with PAP (*B* = 0.094, *p* < 0.001), indicating that the negative association between PEB and PAP became weaker as CSFA increased. Further analysis showed that the Index of Moderated Mediation was significant (Index = 0.007, 95% CI 0.004–0.010). It should be noted that, in the primary Model 7, the Y-model with BADL as the outcome was estimated using OLS. Therefore, the b path, c′ path, conditional indirect effects, and the Index of Moderated Mediation all inherit the limitations associated with OLS modeling under the pronounced ceiling effect in BADL. When Model 7 was re-estimated using dichotomized BADL with a logistic Y-model, the Index of Moderated Mediation remained significant (Index = 0.020, 95% bootstrap CI 0.010–0.032), indicating that the main statistical inference was robust to the alternative specification of the BADL outcome and supporting the inference from the primary analysis.

To assess the robustness of the linear specification of CSFA, CSFA was further treated as a three-category variable and dummy-coded. As shown in Table 10, the overall categorical interaction was significant (Δ*R*^2^ = 0.0050, *F* (2, 5015) = 18.753, *p* < 0.001), and the negative association between PEB and PAP progressively weakened as CSFA increased, with significant differences in slopes across categories. These findings indicate that the moderation pattern was not driven solely by the contrast between CSFA = 0 and CSFA = 1, but should not be interpreted as a strictly equally spaced linear dose–response relationship.

## 5. Discussion

### 5.1. Interpretation in Relation to Prior Research

This study found that PEB was significantly and negatively associated with BADL, which is generally consistent with previous research on physical activity barriers and functional health among older adults. The healthy aging framework emphasizes that older adults’ health status is determined not only by disease burden and physical functioning but also by the behavioral opportunities and environmental conditions required to maintain daily activity capacity [4,5]. Previous longitudinal studies have further shown that PAP levels are closely associated with BADL status among older adults, with higher levels of physical activity participation generally being associated with better functional performance [23]. However, PAP among older adults is not determined solely by individual willingness but is influenced by multiple factors, including physical conditions, risk perceptions, social support, and environmental resources. Relevant systematic reviews have indicated that barriers to PAP among older adults are multidimensional, with physical limitations, concerns about exercise-related risks, insufficient support, and inadequate environmental conditions potentially restricting participation opportunities [10,16]. In addition, studies focusing on older women have suggested that gender roles, health constraints, and differences in social resources may further intensify the constraints associated with PAP [19]. Based on this perspective, the present study incorporated PEB into the analytical framework of functional health among older women. The findings showed that higher PEB was associated not only with lower PAP but also with lower BADL. Notably, although the association between PEB and BADL reached statistical significance, the magnitude of the association was relatively limited. Specifically, a one-standard-deviation increase in PEB was associated with a 0.103-standard-deviation decrease in BADL, suggesting that PEB should be considered one of the factors associated with functional health among older women rather than a single determinant of functional status. Its clinical relevance remains unclear. Future studies should further consider individual physical conditions, social support, and community environments, and use longitudinal or intervention designs to evaluate the practical significance of this association.

This study further found a significant but relatively small indirect association between PEB and BADL through PAP. This finding is consistent with previous research regarding the relationship between physical activity and functional capacity among older adults [7,25]. Previous studies have shown that sustained participation in physical activity is associated with the maintenance of cardiorespiratory fitness, muscle strength, balance ability, and social participation among older adults, and may therefore contribute to delaying functional decline [42,43]. More importantly, the present study further suggests that PAP may represent a behavioral component linking PEB and functional health. Compared with previous studies that have primarily focused on the direct association between physical activity participation levels and health outcomes [23,40], this study further incorporated PEB into the analytical framework, and a sensitivity analysis using dichotomized BADL with a logistic Y-model also supported the main inference regarding this indirect association, suggesting that individuals’ perceptions of exercise participation conditions and constraints may be simultaneously associated with differences in PAP and functional health. PEB may be associated with differences in individual behavioral patterns. When older women continuously perceive barriers such as physical discomfort, lack of time, caregiving responsibilities, or limited environmental conditions, their frequency, duration, and level of engagement in physical activity may decrease accordingly [44]. Theoretically, such behavioral changes may first manifest as reduced PAP; however, persistently low levels of physical activity may reduce the activity stimuli required to maintain physical functioning and may further be associated with a higher risk of functional limitations [45,46]. In other words, PAP may represent a behavioral component in the association between PEB and functional health [9].

This study further found that the negative association between PEB and PAP became weaker as CSFA increased, and that the absolute magnitude of the corresponding conditional indirect association decreased. This finding is consistent with the perspective of social ecological theory, which suggests that health behaviors are shaped not only by individual perceptions but also by external environmental conditions [7,25]. Previous studies have generally indicated that age-friendly environments are closely associated with physical activity levels among older adults [28,29]. The present study further highlights how community resource conditions may shape the association between PEB and insufficient PAP. CSFA may play a conditional role because it can create more opportunities for participation among older women by reducing participation costs and improving accessibility and convenience [30]. The categorical robustness analysis further supported this pattern, indicating that the moderating relationship was not driven solely by differences between the lower-resource groups. In addition, the sensitivity analysis using dichotomized BADL with a logistic Y-model also supported the main inference. Overall, community sports facility resources may buffer the association between individual barriers and PAP to some extent, but cannot fully substitute for the roles of individual capabilities, family support, and health management.

The subgroup analyses further showed that the negative association between PEB and BADL was directionally consistent across subgroups stratified by age, chronic disease burden, income, educational attainment, household registration type, and marital status, suggesting that this association was not restricted to a specific subgroup. Previous studies have consistently demonstrated that age, chronic disease burden, and socioeconomic resource conditions are associated with physical activity participation, maintenance of health behaviors, and functional health among older adults [13,47]. Subgroup regression analyses showed that the absolute values of the regression coefficients increased with age, with the largest absolute coefficient observed among participants aged 80 years and above. Regarding income groups, the absolute value of the negative coefficient was also relatively larger among participants with an annual income below 2000 yuan. Previous studies have suggested that older adults at advanced ages may experience declines in physical functioning and mobility limitations [48], whereas individuals with lower incomes may face resource constraints related to transportation accessibility, rehabilitation support, and access to appropriate exercise services [49]. However, the formal interaction tests in this study did not identify significant differences between age groups, and the overall interaction effect across income groups did not reach statistical significance. Therefore, these findings only reflect descriptive differences and are insufficient to support clear age- or income-related heterogeneity. The negative coefficients were also numerically larger among participants with lower educational attainment, agricultural household registration, and no spouse. However, because formal interaction tests were not conducted for these subgroup comparisons, these differences should only be interpreted as descriptive evidence. Overall, the negative association between PEB and BADL was directionally consistent across subgroups, whereas whether meaningful differences exist between population groups requires further investigation.

### 5.2. Theoretical and Practical Implications

This study has several theoretical implications. By incorporating PEB, PAP, CSFA, and BADL into the same analytical framework, this study extends the application of social ecological theory in research on physical activity participation and functional health among older women. The findings indicate that the association between PEB and functional health involved not only a direct component but also a significant yet relatively small indirect association through PAP. Meanwhile, CSFA was associated with variations in the strength of the association between PEB and PAP. Accordingly, PAP was identified as a behavioral component linking individual constraints and functional health, while CSFA was characterized as an important environmental condition associated with this pattern of associations. These findings specify the general understanding of the relationships among individual factors, behaviors, and environmental conditions within the social–ecological framework, providing new empirical evidence for understanding how community resources are related to physical activity participation and health outcomes among older women.

This study also has practical implications for community sports governance and health promotion among older women. First, community sports governance should not focus solely on encouraging older women to actively participate in exercise but should also address the practical barriers they encounter during participation. By optimizing facility provision, reducing participation barriers, and improving service support, health promotion strategies may shift from individual behavior-oriented advocacy toward an integrated governance approach that considers both individual needs and environmental support. Second, community sports services should not only improve the accessibility, convenience, and age-friendliness of sports facilities, so that sports resources can be transformed into usable opportunities for health promotion among older women, but should also respond to their practical needs related to caregiving responsibilities, time arrangements, and social support. Flexible scheduling of activities, diversified organizational formats, group-based activities, neighborhood mutual assistance, and accompanied exercise programs may provide additional social support for sustained participation in physical activity. Furthermore, the subgroup analyses indicated that the negative association between perceived exercise barriers and functional health appeared to be more pronounced among some older women with fewer socioeconomic and health resources, although these patterns require further confirmation. Therefore, community-based exercise promotion programs may benefit from considering the diversity of barriers experienced by older women rather than adopting a uniform approach. Efforts to improve exercise accessibility could involve strengthening coordination among community sports facilities, primary healthcare providers, and social support networks to provide more inclusive and supportive opportunities for older adults to engage in physical activity.

### 5.3. Limitations and Future Research

This study has several limitations. First, the cross-sectional design precludes establishing temporal order or causal relationships among PEB, PAP, and BADL. Reverse causation is possible, as lower PAP or poorer BADL may lead respondents to report more PEB, and residual confounding cannot be excluded. Longitudinal or quasi-experimental studies are needed to clarify the temporal ordering of these associations.

Second, the study focused on older women in China, which may limit the generalizability of the findings to other populations. In addition, PAP was constructed from three CLASS items with unequal response ranges and therefore reflects relative activity levels within the sample rather than a calibrated physical activity dose. Findings involving PAP should accordingly be interpreted with caution. Future studies could include gender comparisons and use validated or objectively measured physical activity indicators.

Third, complete-case analysis was used to address missing data. Differences between the included and excluded groups suggest that selection bias cannot be ruled out, and its direction and magnitude remain uncertain because alternative missing-data approaches were not examined. Future research may compare the results obtained using multiple imputation or other missing-data strategies.

Finally, all focal variables were self-reported by the same respondents in the same survey wave and may therefore be affected by recall bias, social desirability bias, and same-source measurement bias. CSFA reflected perceived community facility provision rather than objectively measured accessibility, quality, or usability. Moreover, because CSFA was measured as a 0–2 resource count and treated as a linear moderator in the primary model, the findings should not be interpreted as a strict linear dose–response relationship, and estimates at the highest resource level should be interpreted cautiously. Because the secondary dataset did not include a prespecified marker variable, common method bias could not be statistically adjusted. The reported direct, indirect, and interaction associations should therefore not be interpreted as evidence of causal mechanisms. Future studies should incorporate objective, multi-source, and longitudinal measures.

## 6. Conclusions

Based on data from the 2023 China Longitudinal Aging Social Survey (CLASS), this study examined the associations among Perceived Exercise Barriers (PEB), Physical Activity Participation (PAP), Community Sports Facility Accessibility (CSFA), and Basic Activities of Daily Living (BADL) among older women in China. The results showed that PEB was significantly and negatively associated with BADL, with a significant but relatively small indirect association through PAP. In addition, the strength of the association between PEB and PAP varied across different levels of CSFA. These findings suggest that functional health among older women is significantly associated with individual physical activity participation and community environmental resources. By integrating individual barrier perceptions, behavioral participation, and community environmental conditions, this study extends the understanding of factors associated with functional health among older women and provides empirical evidence and practical implications for developing supportive healthy aging environments and improving quality of life among older women.

## Figures and Tables

**Table 1:** Descriptive statistics for categorical variables (N = 5027).

Variables	Description	Frequency	Percentage
CSFA	No sports resources	2019	40.16%
	Has 1 type of sports resource	2628	52.28%
	Has 2 types of sports resources	380	7.56%
Educational attainment	Illiterate	1137	22.62%
	Private school/literacy class	183	3.64%
	Primary school	1903	37.86%
	Junior high school	1278	25.42%
	Senior high school/technical secondary school	436	8.67%
	Junior college	76	1.51%
	Bachelor’s degree and above	14	0.28%
Marital status	No spouse	1119	22.26%
	With spouse	3908	77.74%
Household registration type	Agricultural household registration	2908	57.85%
	Non-agricultural household registration	2119	42.15%
Individual annual income	≤2000 yuan	288	5.73%
	2000–6000 yuan	1303	25.92%
	6000–10,000 yuan	524	10.42%
	≥10,000 yuan	2912	57.93%

**Table 2:** Descriptive statistics for continuous variables (N = 5027).

Variables	Mean	SD	MIN	MAX
BADL	32.61	1.61	11	33
PEB	3.18	3.98	0	18
PAP	6.14	3.74	3	16
Number of chronic diseases	1.94	1.45	0	14
Age	71.48	6.15	60	101

**Table 3:** Correlation analysis (N = 5027).

Variables	PEB	BADL	PAP	CSFA
PEB	1			
BADL	−0.105 ***	1		
PAP	−0.386 ***	0.091 ***	1	
CSFA	−0.035 *	−0.029 *	0.121 ***	1

* p < 0.05, *** *p* < 0.001.

**Table 4:** Sociodemographic Characteristics and BADL Outcomes by PAP Level.

Variable	Category/Statistic	Lower PAP Group (n = 2609)	Higher PAP Group (n = 2418)	Test Statistic	*p*
Panel A. Group Comparisons of Sociodemographic Characteristics and BADL Outcomes					
Educational attainment	Illiterate	690 (26.4%)	447 (18.5%)	*χ*^2^ = 150.606	<0.001
	Private school/literacy class	104 (4.0%)	79 (3.3%)		
	Primary school	1081 (41.4%)	822 (34.0%)		
	Junior high school	539 (20.7%)	739 (30.6%)		
	Senior high school/technical secondary school	161 (6.2%)	275 (11.4%)		
	Junior college	30 (1.1%)	46 (1.9%)		
	Bachelor’s degree and above	4 (0.2%)	10 (0.4%)		
Marital status	No spouse	659 (25.3%)	460 (19.0%)	*χ*^2^ = 28.190	<0.001
	With spouse	1950 (74.7%)	1958 (81.0%)		
Household registration type	Agricultural household registration	1797 (68.9%)	1111 (45.9%)	*χ*^2^ = 270.594	<0.001
	Non-agricultural household registration	812 (31.1%)	1307 (54.1%)		
Personal annual income	≤2000 yuan	167 (6.4%)	121 (5.0%)	*χ*^2^ = 25.715	<0.001
	2000–6000 yuan	742 (28.4%)	561 (23.2%)		
	6000–10,000 yuan	262 (10.0%)	262 (10.8%)		
	≥10,000 yuan	1438 (55.1%)	1474 (61.0%)		
BADL	Median (IQR)	33.00 (0.00)	33.00 (0.00)	*U* = 2,995,748	<0.001
Panel B. Multivariable Logistic Regression for Predicting BADL Independence					
PAP group	Lower PAP group	Reference			
	Higher PAP group		aOR = 1.734 95% CI 1.437–2.093		<0.001

Categorical variables are presented as n (%), and group differences were examined using Pearson’s *χ*^2^ tests. BADL is presented as median (IQR), and group differences were assessed using the Mann–Whitney *U* test. In the binary logistic regression analysis, a BADL score of 33 points was defined as “complete independence” (1), while scores below 33 points were defined as “functional limitations” (0). The lower PAP group was used as the reference group, and the model was adjusted for age, number of chronic diseases, educational attainment, marital status, household registration type, and personal annual income. aOR, adjusted odds ratio.

**Table 5:** Results of hierarchical OLS regression analysis.

Variables	BADL (1)	BADL (2)
PEB	−0.104 ***	−0.103 ***
	(0.014)	(0.014)
Age		−0.017 ***
		(0.003)
Number of chronic diseases		−0.050 ***
		(0.010)
Educational attainment		0.005
		(0.013)
Marital status		0.003
(ref.: no spouse)		(0.036)
Household registration type		−0.017
(ref.: agricultural)		(0.033)
Individual annual income		0.030 *
		(0.014)
_cons	0.000	1.202 ***
	(0.014)	(0.214)
N	5027	5027
*R* ^2^	0.011	0.031
Adjusted *R*^2^	0.011	0.029
*F*	55.438 ***	22.716 ***

PEB and BADL were standardized variables. In the fully adjusted model (Model 2), the VIF values for all predictors ranged from 1.041 to 1.403. Additional details are provided in Supplementary Material S3. * *p* < 0.05, *** *p* < 0.001.

**Table 6:** Results of the additional analysis with self- rated health as the outcome.

Variables	Self-Rated Health (1)	Self-Rated Health (2)
PEB	−0.219 ***	−0.205 ***
	(0.011)	(0.011)
Age		−0.014 ***
		(0.002)
Number of chronic diseases		−0.116 ***
		(0.007)
Educational attainment		0.018
		(0.009)
Marital status		0.078 **
(ref.: no spouse)		(0.027)
Household registration type		0.140 ***
(ref.: agricultural)		(0.024)
Individual annual income		0.032 **
		(0.011)
_cons	3.496 ***	4.416 ***
	(0.011)	(0.158)
N	5027	5027
*R* ^2^	0.076	0.155
Adjusted *R*^2^	0.076	0.154
*F*	414.862 ***	131.731 ***

** *p* < 0.01, *** *p* < 0.001.

**Table 7:** Subgroup Analysis Results.

**(a)**				
	**(1)**	**(2)**		**(3)**
	**60–69 Years**	**70–79 Years**		**80 Years and Above**
PEB	−0.084 *** (0.018)	−0.097 *** (0.020)		−0.199 ** (0.076)
Other controls	Yes	Yes		Yes
N	2007	2443		577
*R* ^2^	0.018	0.020		0.042
PEB × Age group: test of between-group differences	Reference	−0.000 (0.029)		−0.059 (0.051)
Interaction *p* value		0.991		0.243
Joint interaction test	*F*(2, 5016) = 0.75, *p* = 0.471			
**(b)**				
	**(1)**	**(2)**		**(3)**
	**No Chronic Disease**	**One Chronic Disease**		**Multiple Chronic Diseases**
PEB	−0.110 ** (0.038)	−0.090 *** (0.026)		−0.109 *** (0.019)
Other controls	Yes	Yes		Yes
N	803	1385		2839
*R* ^2^	0.020	0.026		0.037
**(c)**				
	**(1)**	**(2)**	**(3)**	**(4)**
	**Below 2000 RMB**	**2000–6000 RMB**	**6000–10,000 RMB**	**Above 10,000 RMB**
PEB	−0.215 * (0.094)	−0.129 *** (0.034)	−0.122 * (0.060)	−0.082 *** (0.014)
Other controls	Yes	Yes	Yes	Yes
N	288	1303	524	2912
*R* ^2^	0.047	0.026	0.014	0.046
PEB × Income group: test of between-group differences	−0.143 * (0.063)	−0.058 (0.034)	−0.058 (0.049)	Reference
Interaction *p* value	0.023	0.090	0.238	
Joint interaction test	*F*(3, 5014) = 2.52, *p* = 0.056			
**(d)**				
	**(1)**	**(2)**		**(3)**
	**Below Primary School**	**Primary School**		**Junior High School and Above**
PEB	−0.160 *** (0.040)	−0.107 *** (0.023)		−0.076 ** (0.017)
Other controls	Yes	Yes		Yes
N	1320	1903		1804
*R* ^2^	0.040	0.017		0.047
**(e)**				
	**(1)**		**(2)**	
	**Agricultural Household Registration**		**Non-Agricultural Household Registration**	
PEB	−0.150 *** (0.026)		−0.071 *** (0.013)	
Other controls	Yes		Yes	
N	2908		2119	
*R* ^2^	0.020		0.105	
**(f)**				
	**(1)**		**(2)**	
	**No Spouse**		**With Spouse**	
PEB	−0.113 ** (0.040)		−0.098 *** (0.015)	
Other controls	Yes		Yes	
N	1119		3908	
*R* ^2^	0.043		0.026	

* *p* < 0.05, ** *p* < 0.01, *** *p* < 0.001.

**Table 8:** Results of Indirect Association Analysis (PROCESS Model 4).

**Variables**	**PAP (M)**				**BADL (Y)**			
	**Coeff.**	**SE**	**LLCI**	**ULCI**	**Coeff.**	**SE**	**LLCI**	**ULCI**
PAP (M)					0.072 ***	0.017	0.039	0.105
PEB (X)	−0.471 ***	0.012	−0.494	−0.447	−0.069 ***	0.016	−0.102	−0.037
Age	−0.007 ***	0.002	−0.011	−0.003	−0.016 ***	0.003	−0.022	−0.011
Number of chronic diseases	−0.050 ***	0.008	−0.066	−0.034	−0.047 ***	0.010	−0.066	−0.027
Educational attainment	0.038 ***	0.011	0.018	0.059	0.002	0.013	−0.022	0.027
Marital status	0.397 ***	0.027	0.343	0.450	−0.045	0.033	−0.110	0.020
Household registration type	−0.030 *	0.012	−0.054	−0.007	0.033 *	0.014	0.004	0.061
Income	0.051	0.030	−0.008	0.110	0.000	0.036	−0.071	0.070
Constant	0.386 *	0.179	0.035	0.737	1.174 ***	0.213	0.756	1.592
*R* ^2^	0.320 ***				0.034 ***			
**Indirect effects of X on Y**	**Effect**		**Boot SE**		**Boot LLCI**		**Boot ULCI**	
Primary analysis: continuous BADL, OLS Y-model	−0.034		0.005		−0.044		−0.024	
Sensitivity analysis: binary BADL, logistic Y-model	−0.098		0.024		−0.147		−0.051	

X = Perceived Exercise Barriers index; M = Physical activity index; Y = Basic Activities of Daily Living score. In the dichotomized BADL sensitivity analysis, 1 indicates complete independence and 0 indicates functional limitation. The indirect effect estimated using the logistic model is expressed on the log-odds scale and therefore should not be directly compared with the effect estimate from the primary analysis. * *p* < 0.05, *** *p* < 0.001.

**Table 9:** Results of Conditional Indirect Association Analysis (PROCESS Model 7).

**Variables**	**PAP (M)**				**BADL (Y)**			
	**Coeff.**	**SE**	**LLCI**	**ULCI**	**Coeff.**	**SE**	**LLCI**	**ULCI**
PAP (M)					0.072 ***	0.017	0.039	0.105
PEB (X)	−0.476 ***	0.012	−0.500	−0.453	−0.069 ***	0.016	−0.102	−0.037
CSFA (W)	0.171 ***	0.019	0.133	0.209				
X × W	0.094 ***	0.018	0.059	0.130				
Age	−0.007 **	0.002	−0.011	−0.003	−0.016 ***	0.003	−0.022	−0.011
Number of chronic diseases	−0.054 ***	0.008	−0.070	−0.038	−0.047 ***	0.010	−0.066	−0.027
Educational attainment	0.030 **	0.010	0.009	0.050	0.002	0.013	−0.022	0.027
Marital status	0.368 ***	0.027	0.315	0.421	−0.045	0.033	−0.110	0.020
Household registration type	−0.022	0.012	−0.046	0.001	0.033 *	0.014	0.004	0.061
Income	0.056	0.030	−0.003	0.114	−0.000	0.036	−0.071	0.070
Constant	0.391 *	0.177	0.044	0.739	1.174 ***	0.213	0.756	1.592
*R* ^2^	0.333 ***				0.034 ***			
**CSFA Level**	**Effect**		**Boot SE**		**Boot LLCI**		**Boot ULCI**	
CSFA = 0 (No sports resource type)	−0.0387		0.0057		−0.0502		−0.0278	
CSFA = 1 (One sports resource type)	−0.0319		0.0047		−0.0416		−0.0231	
CSFA = 2 (Two sports resource types)	−0.0251		0.0040		−0.0336		−0.0178	
**Index of Moderated Mediation**	**Index**		**Boot SE**		**Boot LLCI**		**Boot ULCI**	
Primary analysis: continuous BADL, OLS Y-model	0.0068		0.0014		0.0042		0.0099	
Sensitivity analysis: binary BADL, logistic Y-model	0.0195		0.0057		0.0097		0.0319	

BADL, PEB, and PAP were standardized using Z-score transformation. CSFA retained its original 0–2 scoring format and was mean-centered only for the interaction term analysis. The number of bootstrap resamples was set at 5000. In the primary analysis, the BADL Y-model was estimated using OLS; in the sensitivity analysis, BADL was dichotomized and the Y-model was estimated using logistic regression. Because the two models are based on different effect scales, the magnitudes of the indices were not directly compared. * *p* < 0.05, ** *p* < 0.01, *** *p* < 0.001.

**Table 10:** Robustness Test of the Moderating Effect of PEB–PAP Using Dummy-Coded CSFA.

Item	*B*	*SE*	*t*	*p*	95% CI
**Panel A. Conditional Effects of PEB on PAP at Different Levels of CSFA**					
CSFA = 0 (No sports resource type)	−0.537	0.020	−27.081	<0.001	[−0.576, −0.498]
CSFA = 1 (One sports resource type)	−0.454	0.016	−28.189	<0.001	[−0.485, −0.422]
CSFA = 2 (Two sports resource types)	−0.292	0.036	−8.188	<0.001	[−0.362, −0.222]
**Panel B. Between-Category Differences in PEB–PAP Slopes**					
CSFA = 1 vs. CSFA = 0	0.083	0.025	3.314	<0.001	[0.034, 0.133]
CSFA = 2 vs. CSFA = 0	0.246	0.041	6.017	<0.001	[0.166, 0.325]
CSFA = 2 vs. CSFA = 1	0.162	0.039	4.150	<0.001	[0.086, 0.239]

## Data Availability

The CLASS data are available at http://class.ruc.edu.cn/English/Home.htm (accessed on 1 September 2025).

## Supplementary Materials

The following supporting information can be downloaded at: https://www.mdpi.com/article/10.3390/healthcare14172832/s1, S1: STROBE Checklist. S2: Missing Data and Observable Characteristics Comparison. S3: OLS Regression Model Diagnostic Tests.

## Abbreviations

The following abbreviations are used in this manuscript:

CLASS	China Longitudinal Aging Social Survey
BADL	Basic Activities of Daily Living
PEB	Perceived Exercise Barriers
PAP	Physical Activity Participation
CSFA	Community Sports Facility Accessibility
