# Supplementary material for: Perceived Exercise Barriers and Functional Health Among Older Chinese Women: The Roles of Physical Activity Participation and Community Sports Facility Accessibility
Source: Healthcare (Basel). 2026 Sep 3;14(17):2832. doi: 10.3390/healthcare14172832 (PMC13566427; doi:10.3390/healthcare14172832)
Supplement: Supplementary file 1 [file healthcare-14-02832-s001.zip › Supplementary Material S1_STROBE Checklist.pdf]

STROBE Statement—checklist of items that should be included in reports of observational studies

	Item No.	Recommendation	Page/Position	Note
**Title and abstract**	1	(*a*) Indicate the study's design with a commonly used term in the title or the abstract	p1-2	"Cross-sectional study" is added in the Abstract Methods section; Section 3.1 clearly states that data from the 2023 China Longitudinal Aging Social Survey (CLASS) were used to conduct a cross-sectional study.
		(*b*) Provide in the abstract an informative and balanced summary of what was done and what was found	p2	Corresponding content is provided in the Abstract.
**Introduction**				
Background/rationale	2	Explain the scientific background and rationale for the investigation being reported	p3 – 4	Corresponding content is provided in the Introduction.
Objectives	3	State specific objectives, including any prespecified hypotheses	p3 – 4	Corresponding content is provided in the Introduction.
**Methods**				
Study design	4	Present key elements of study design early in the paper	p7	Corresponding content is provided in Section 3.1.
Setting	5	Describe the setting, locations, and relevant dates, including periods of recruitment, exposure, follow-up, and data collection	N/A	N/A; publicly available database.
Participants	6	(*a*) Give the eligibility criteria, and the sources and methods of selection of participants	p7	Corresponding content is provided in Section 3.1.
Variables	7	Clearly define all outcomes, exposures, predictors, potential confounders, and effect modifiers. Give diagnostic criteria, if applicable	p7 – 9	Corresponding content is provided in Section 3.2.
Data sources/ measurement	8*	For each variable of interest, give sources of data and details of methods of assessment (measurement). Describe comparability of assessment methods if there is more than one group	p6 – 9	*Corresponding content is provided in Section 3.*
Bias	9	Describe any efforts to address potential sources of bias	p9 – 11	Corresponding content is provided in Section 3.3.
Study size	10	Explain how the study size was arrived at	p7	Corresponding content is provided in Section 3.1.
Quantitative variables	11	Explain how quantitative variables were handled in the	p7 – 9	Corresponding content is provided in Section 3.2.

		analyses. If applicable, describe which groupings were chosen and why		
Statistical methods	12	(*a*) Describe all statistical methods, including those used to control for confounding	p9 – 11	Corresponding content is provided in Section 3.3.
		(*b*) Describe any methods used to examine subgroups and interactions	p9 – 11	Corresponding content is provided in Section 3.3.
		(*c*) Explain how missing data were addressed	p9 – 11	Corresponding content is provided in Section 3.3.
		(*d*) If applicable, describe analytical methods taking account of sampling strategy	N/A	N/A; publicly available database.
		(*e*) Describe any sensitivity analyses	p9 – 11,	Corresponding content is provided in Section 3.3.
**Results**				
Participants	13*	(a) Report numbers of individuals at each stage of study—eg numbers potentially eligible, examined for eligibility, confirmed eligible, included in the study, completing follow-up, and analysed	p11	Corresponding content is provided in Section 4.1.
		(b) Give reasons for non-participation at each stage	p11	Corresponding content is provided in Section 4.1.
		(c) Consider use of a flow diagram	p11	The participant selection process is fully reported in text in Section 4.1.
Descriptive data	14*	(a) Give characteristics of study participants (eg demographic, clinical, social) and information on exposures and potential confounders	p11 – 12, Tables 1 and 2	Corresponding content is provided in Tables 1 and 2.
		(b) Indicate number of participants with missing data for each variable of interest	p11	Corresponding content is provided in Section 4.1.
Outcome data	15*	Cross-sectional study—Report numbers of outcome events or summary measures	p11 – 15	Corresponding content is provided in Sections 4.1 – 4.3.
Main results	16	(a) Give unadjusted estimates and, if applicable, confounder-adjusted estimates and their precision (eg, 95% confidence interval). Make clear which confounders were adjusted for and why they were included	p13 – 15	Corresponding content is provided in Sections 4.1 and 4.2.
		(b) Report category boundaries when continuous variables were categorized	p11 – 14	Corresponding content is provided in Sections 4.2 and 4.5.
		(c) If relevant, consider translating estimates of relative risk into absolute risk for a meaningful time period	N/A	N/A.

Other analyses	17	Report other analyses done—eg analyses of subgroups and interactions, and sensitivity analyses	p13; P16-21	Corresponding content is provided in Sections 4.2 and 4.5 – 4.7.
**Discussion**				
Key results	18	Summarise key results with reference to study objectives	p21-23	Corresponding content is provided in Sections 5.1.
Limitations	19	Discuss limitations of the study, taking into account sources of potential bias or imprecision. Discuss both direction and magnitude of any potential bias	p24-25	Corresponding content is provided in Section 5.3.
Interpretation	20	Give a cautious overall interpretation of results considering objectives, limitations, multiplicity of analyses, results from similar studies, and other relevant evidence	p21-23; p24-25	Corresponding content is provided in Sections 5.1 and 5.3.
Generalisability	21	Discuss the generalisability (external validity) of the study results	p24-25	Corresponding content is provided in Section 5.3.
**Other information**				
Funding	22	Give the source of funding and the role of the funders for the present study and, if applicable, for the original study on which the present article is based	p26	Corresponding content is provided in the Funding section.
