# Supplementary material for: Perceived Exercise Barriers and Functional Health Among Older Chinese Women: The Roles of Physical Activity Participation and Community Sports Facility Accessibility
Source: Healthcare (Basel). 2026 Sep 3;14(17):2832. doi: 10.3390/healthcare14172832 (PMC13566427; doi:10.3390/healthcare14172832)
Supplement: Supplementary file 1 [file healthcare-14-02832-s001.zip › Supplementary Material S2_Missing Data and Observable Characteristics Comparison.pdf]

**Supplementary Material S2:** Comparison of Observable Characteristics and Missing Data Between the Complete-Case Group and Excluded Group

**Panel A. Missing Data and Formation of the Analytical Sample**

| Item | n | Percentage of Eligible Sample (%) | Description |
|---|---|---|---|
| Female respondents meeting age and sex eligibility criteria | 5606 | 100.00 | Reference sample for missing data assessment |
| Missing personal annual income | 576 | 10.27 | Among them, 573 had missing income only, and 3 had missing income and self-rated health simultaneously |
| Missing self-rated health information | 6 | 0.11 | Among them, 3 had missing self-rated health only, and 3 had missing self-rated health and personal annual income simultaneously |
| Missing both variables | 3 | 0.05 | Overlapping cases with missing personal annual income and self-rated health |
| Excluded due to missing values in either variable | 579 | 10.33 | Complete-case analysis was applied |
| Final complete-case analytical sample | 5027 | 89.67 | No missing values were observed in other major study variables |
| Missing values in other key study variables or covariates | 0 | 0.00 | Including PEB, PAP, BADL, CSFA, and other covariates included in the analysis |

**Panel B. Comparison of Observable Characteristics Between the Excluded Group and Complete-Case Group**

| Variable | Excluded group (n=579） | Complete-case group (n=5027) | Test statistic | p value | Effect size |
|---|---|---|---|---|---|
| Age (years) | 70.04 ± 5.31 | 71.48 ± 6.15 | Welch t = -6.034 | <0.001 | Cohen's d = -0.236 |
| Number of chronic diseases | 0.90 ± 1.21 | 1.94 ± 1.45 | Welch t = -19.214 | <0.001 | Cohen's d = -0.727 |
| Educational attainment | | | $\chi^2$ = 276.119, df = 6 | <0.001 | Cramér's V = 0.222 |
| Illiterate | 72 (12.4%) | 1137 (22.6%) | | | |
| Private school/literacy class | 10 (1.7%) | 183 (3.6%) | | | |
| Primary school | 133 (23.0%) | 1903 (37.9%) | | | |
| Junior high school | 178 (30.7%) | 1278 (25.4%) | | | |

| | | | | | |
|---|---|---|---|---|---|
| Senior high school/technical secondary school | 170 (29.4%) | 436 (8.7%) | | | |
| Junior college | 13 (2.2%) | 76 (1.5%) | | | |
| Bachelor’s degree and above | 3 (0.5%) | 14 (0.3%) | | | |
| Marital status | | | $\chi^2$ = 22.012, df = 1 | <0.001 | Cramér's V = 0.063 |
| No spouse | 80 (13.8%) | 1119 (22.3%) | | | |
| With spouse | 499 (86.2%) | 3908 (77.7%) | | | |
| Household registration type | | | $\chi^2$ = 28.258, df = 1 | <0.001 | Cramér's V = 0.071 |
| Agricultural household registration | 268 (46.3%) | 2908 (57.8%) | | | |
| Non-agricultural household registration | 311 (53.7%) | 2119 (42.2%) | | | |

[1] Continuous variables are presented as mean ± standard deviation. Welch-adjusted independent-samples t tests were used for comparisons of continuous variables. Categorical variables are presented as n (%), and Pearson’s $\chi^2$ tests were applied for categorical variables. Effect sizes for continuous variables were calculated using Cohen’s d, and effect sizes for categorical variables were calculated using Cramér’s V. The sign of Cohen’s d was calculated as the mean difference between the excluded group and the complete-case group. This comparison was conducted to describe potential selection differences associated with complete-case analysis and does not determine whether the missing data mechanism was Missing Completely At Random (MCAR), Missing At Random (MAR), or Missing Not At Random (MNAR).

Among the 5606 female respondents who met the age and sex eligibility criteria, 576 had missing individual annual income data, and 6 had missing self-rated health information, of whom 3 had missing values for both variables. Therefore, 579 respondents were excluded due to missing information on these variables, resulting in a final analytical sample of 5027 participants based on complete-case analysis. No missing values were observed in other key study variables or covariates.

Group comparisons showed that participants in the complete-case group were older on average and had a greater number of chronic diseases. Significant differences were also observed between the two groups in the distributions of educational attainment, marital status, and household registration type (all $p < 0.001$). Regarding effect sizes, the between-group difference in the number of chronic diseases was relatively large (Cohen’s d = 0.727), while the difference in educational attainment was relatively notable (Cramér’s V = 0.222). In contrast, differences in age, marital status, and household registration type were relatively small. Overall, the complete-case group and excluded group differed in some observable characteristics; therefore, potential selection bias associated with complete-case analysis should be considered when interpreting the findings.
