# Supplementary material for: Perceived Exercise Barriers and Functional Health Among Older Chinese Women: The Roles of Physical Activity Participation and Community Sports Facility Accessibility
Source: Healthcare (Basel). 2026 Sep 3;14(17):2832. doi: 10.3390/healthcare14172832 (PMC13566427; doi:10.3390/healthcare14172832)
Supplement: Supplementary file 1 [file healthcare-14-02832-s001.zip › Supplementary Material S3_OLS Regression Model Diagnostic Tests.pdf]

Supplementary Material S3: Diagnostic Tests for the OLS Regression Model

This diagnostic analysis was conducted for the fully adjusted OLS regression model (Model 2) reported in Table 5 of the main manuscript. The processing of the dependent variable, key independent variable, and covariates was consistent with that used in the primary analyses. Specifically, the model used the Z-score standardized Basic Activities of Daily Living (BADL) score as the dependent variable and the Z-score standardized Perceived Exercise Barriers (PEB) score as the key independent variable, while adjusting for age, number of chronic diseases, educational attainment, marital status, household registration type, and individual annual income. The analytical sample size was 5027.

The collinearity diagnostic results showed that the variance inflation factors (VIFs) of all predictors ranged from 1.041 to 1.403, indicating no substantial multicollinearity. The standardized residuals ranged from −13.505 to 0.863, with a mean of 0.000 and a standard deviation of 0.999.

As shown in Figure S1, both the histogram of standardized residuals and the normal P–P plot indicated substantial deviations from normality in the residual distribution. The scatter plot of standardized residuals against standardized predicted values showed a certain degree of systematic pattern, suggesting limited stability of residual variance. These diagnostic findings were consistent with the pronounced ceiling effect of the BADL variable: among the 5027 respondents, 4437 (88.3%) achieved the maximum score of 33 points. Therefore, the conventional OLS model was not interpreted as fully satisfying the classical regression assumptions, and an upper-censored Tobit model was further applied as a sensitivity analysis in Section 4.3 of the main manuscript.

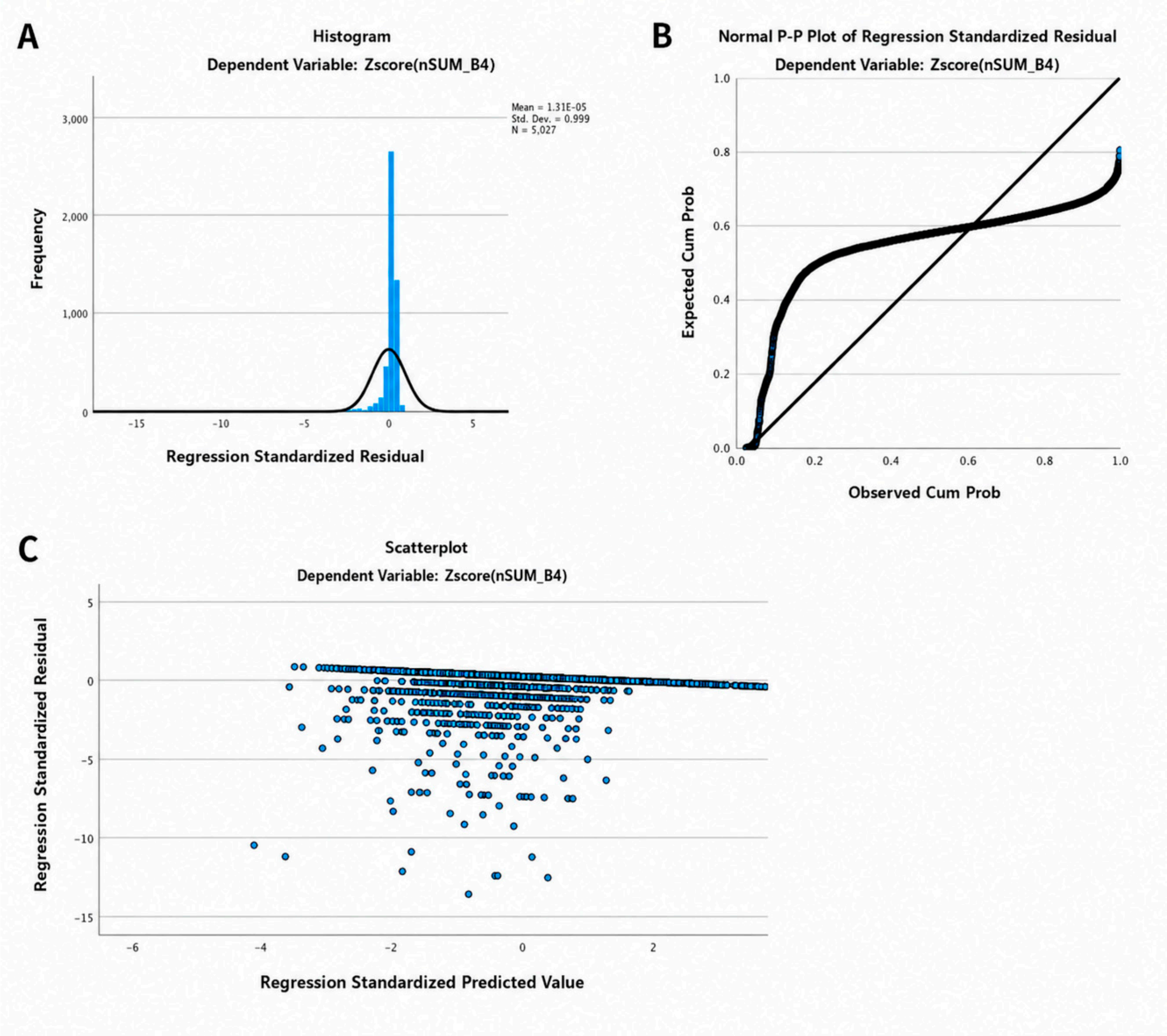

Figure S1. Diagnostic Plots for the OLS Regression Model. (A) Histogram of standardized residuals with an overlaid normal curve; (B) normal P–P plot of standardized residuals; (C) scatter plot of standardized residuals against standardized predicted values. The dependent variable was the Z-score standardized BADL score.
